# Osteolytic Lesions: An Unexpected Diagnosis

**DOI:** 10.7759/cureus.79808

**Published:** 2025-02-28

**Authors:** Pedro Mesquita, Diogo Ferreira da Silva, Sofia Mateus, Teresa Maria Costa, Luís Ferrão Vale

**Affiliations:** 1 Internal Medicine, Unidade Local de Saúde de São José, Hospital de Santo António dos Capuchos, Lisboa, PRT; 2 Surgical Pathology, Unidade Local de Saúde Lisboa Ocidental, Lisboa, PRT

**Keywords:** hypercalcemia, kidney failure, multiple myeloma, osteolytic lesions, primary bone lymphoma

## Abstract

This is the case of a patient who went to the emergency department with back pain and constitutional symptoms (weight loss, anorexia, nausea) lasting one month. Blood tests revealed hypercalcemia and new-onset kidney failure that prompted admission for study. Bone X-ray revealed multiple lytic lesions, and myeloma screening was negative. After a bone marrow biopsy guided by ultrasound of one lesion, it was possible to obtain the diagnosis of diffuse large B-cell lymphoma. We present this case for the diagnostic challenge and atypical presentation simulating multiple myeloma with hypercalcemia.

## Introduction

Primary bone lymphoma (PBL) is a rare entity, consisting of solitary or multifocal osteolytic lesions without evidence of lymph node or extranodal tissue involvement. It represents 7% of malignant bone tumors and 5% of extranodal lymphomas [1].

Bone lymphoma is classified as primary (PBL) or secondary (SBL), distinguished by its origin as either exclusive to the bone or as a result of a disseminated disease with secondary infiltration of the bone. Nevertheless, it is difficult in many cases to distinguish the primary site of lymphoma and to categorize it as PBL or SBL [2].

PBL is one of the rarest malignant bone tumors, representing less than 2% of lymphomas in adults [3]. It manifests as lytic bone lesions, either solitary or multiple, with the most common clinical presentation being localized bone pain (80%-90%), which may be associated with bone fractures (15%-20%) and sometimes with a palpable mass. It can affect any age group, but it is more common in adults aged 45 to 60, with a male predominance [1,4,5].

The criteria for diagnosing this rare entity were described by Coley et al. [6] and are known as Coley’s criteria. They include (a) evidence of primary bone lesion, (b) unequivocal histological confirmation of the bone lesion, and (c) absence of soft tissue involvement or lymphadenopathy.

## Case presentation

This is the case of a 55-year-old male patient with a medical history of type 2 diabetes mellitus under insulin therapy, chronic liver disease of alcoholic etiology, chronic pancreatitis with multiple episodes of exacerbation during the latest years, and still an active smoker with an estimated consumption of 45 pack-years. He was hospitalized on November 2018 for portal vein thrombosis and was followed up in the gastroenterology outpatient clinic of our hospital, with a history of continued alcohol consumption. The previous endoscopic study showed no abnormalities or no varices, and the abdominal ultrasound showed steatosis, portal cavernoma, and cholelithiasis. The patient presented on April 2019 to the emergency department after falling and sustaining pelvic trauma, and accidentally lytic lesions were found in the proximal femur of both legs, and she was referred to orthopedics. At that time, no further investigation was carried out in the emergency setting, and the patient was not followed up.

The patient returned one month later to the emergency department with a consumptive syndrome lasting approximately one month, associated with significant weight loss, localized pain in the lumbar and hip-femoral regions, and difficulty walking. In the emergency work-up, the following findings were noted: normocytic/normochromic anemia, elevated erythrocyte sedimentation rate, new-onset renal dysfunction, severe hypercalcemia, and hypomagnesemia (Table 1). The ECG showed no changes.

**Table 1:** Patient's laboratory results with reference values MCV: mean corpuscular volume; ESR: erythrocyte sedimentation rate; ALP: alkaline phosphatase; LDH: lactate dehydrogenase

	Patient results	Reference values
Hemoglobin	11,1 g/dL	12-15 x 10g/L
MCV	84 fL	82-98 fL
ESR	65 mm/h	0-20 mm/h
Urea	73 mg/dL	16,6-48,5 mg/dL
Creatinine	2,02 mg/dL	0,51-0,95 mg/dL
ALP	223 U/L	35-104 U/L
LDH	258 U/L	135-214 U/L
Calcium	14,4 mg/dL	6,6-10 mg/dL
Ionized calcium (arterial blood)	1,9 mmol/L	1,15-1,29 mmol/L
Magnesium	1,31 mg/dL	1,6-2,6 mg/dL
Phosphorus	4,9 mg/dL	2,5-4,5 mg/dL

**Figure 1:**
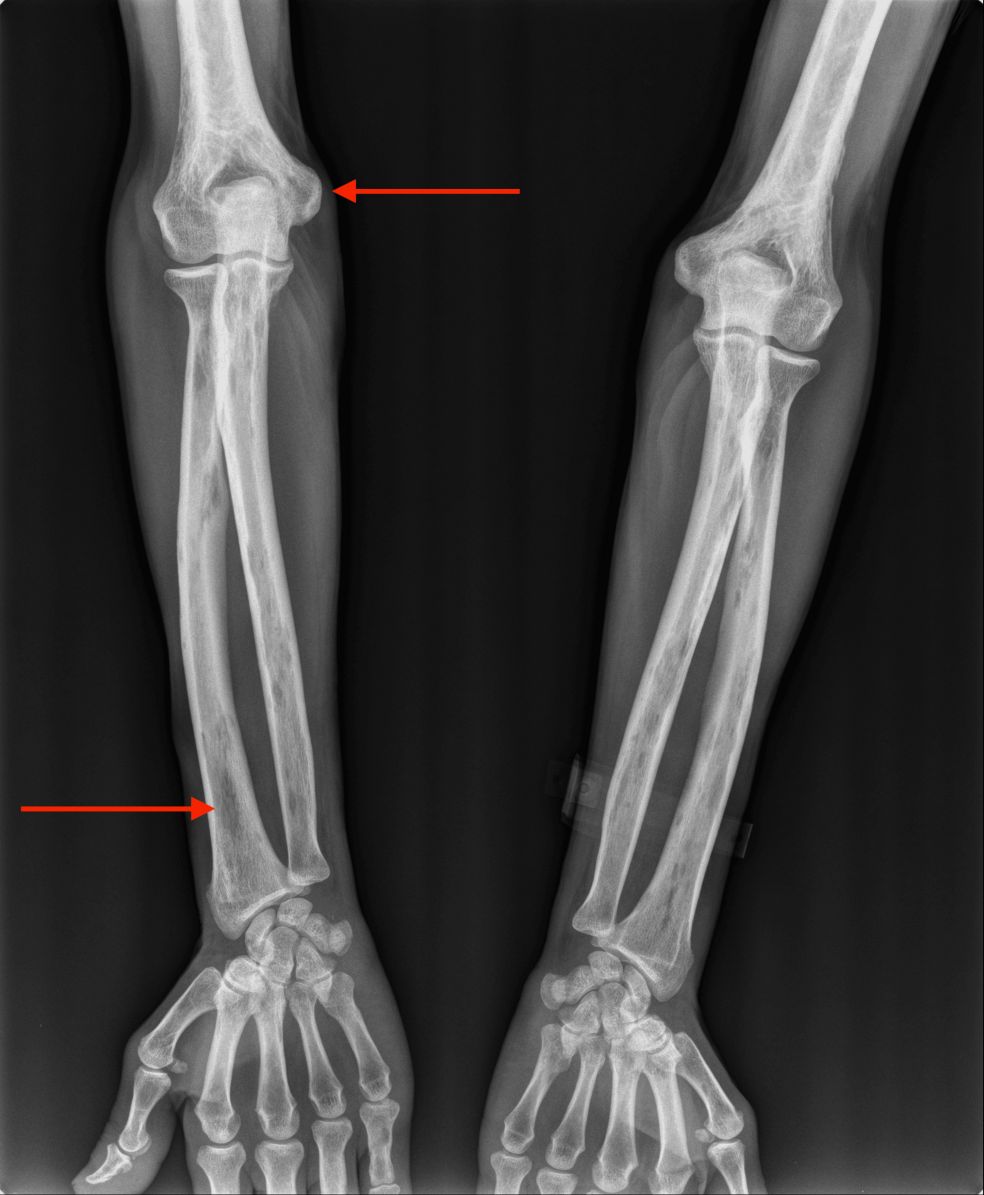
X-ray of the forearm Osteolytic lesions present in the humerus, ulna, and radius (red arrows)

**Figure 2:**
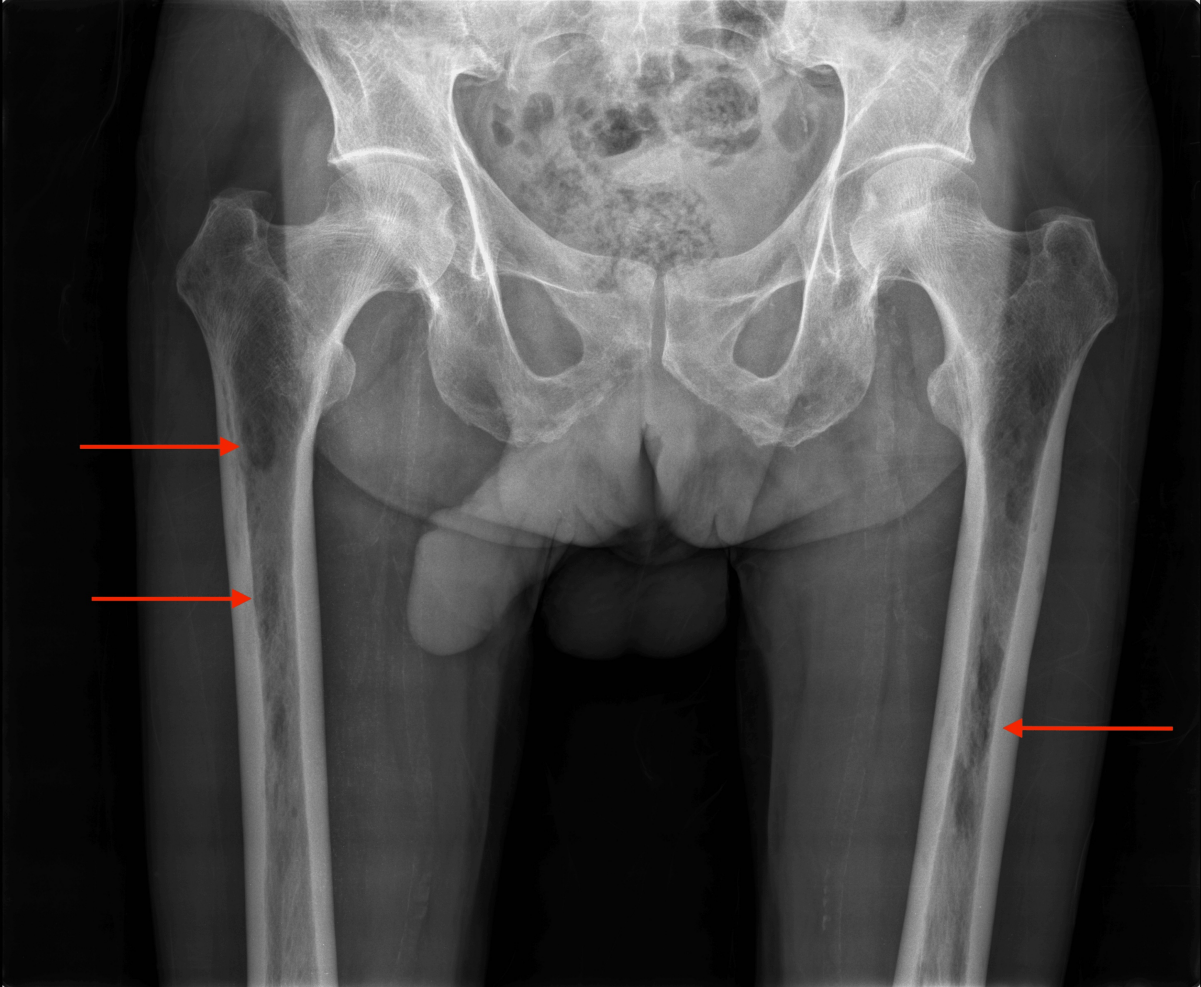
X-ray of the femur Right and left femur with osteolytic lesions (red arrows)

In light of the diagnostic findings, vigorous hydration with 0.9% sodium chloride was initiated, and the patient was treated with a loop diuretic and pamidronate. On the follow-up reevaluation of the laboratory altered values in the emergency department, there was an improvement in renal function and a concurrent resolution of hypercalcemia and hypophosphatemia. The patient was admitted for further clinical investigation. The etiological work-up included a skeletal X-ray, which revealed multiple osteolytic lesions (Figures 1, 2).

Protein electrophoresis showed no evidence of monoclonal peaks, and immunofixation electrophoresis of globulins, Bence-Jones protein in the urine, and free light chain studies were negative. This was complemented by a bone biopsy (not directed at a specific lesion), which showed no alterations. The bone marrow biopsy revealed normal cellularity with 9% lymphocytic series (3% plasma cells; 6% lymphocytes) and 1% blasts, with no evidence of plasmacytic infiltration in the sample.

Continuing the etiological work-up, an abdominal-pelvic CT scan showed no primary thoracic, abdominal, or pelvic tumors but identified osteolytic lesions. Based on the distribution and osteolytic character, multiple myeloma was considered the most probable diagnosis. To exclude central nervous system involvement and assess the extent of osteolytic lesions in the calvaria, a cranial CT scan was performed. No focal parenchymal anomalies were found, but bone alterations were noted, specifically at the right frontal level, with erosion and disruption of the outer table and irregular contour, extending posteriorly to the contour of the frontal sinus; and erosive changes, presumptively significant in the context, in the left zygomatic-malar region.

**Figure 3:**
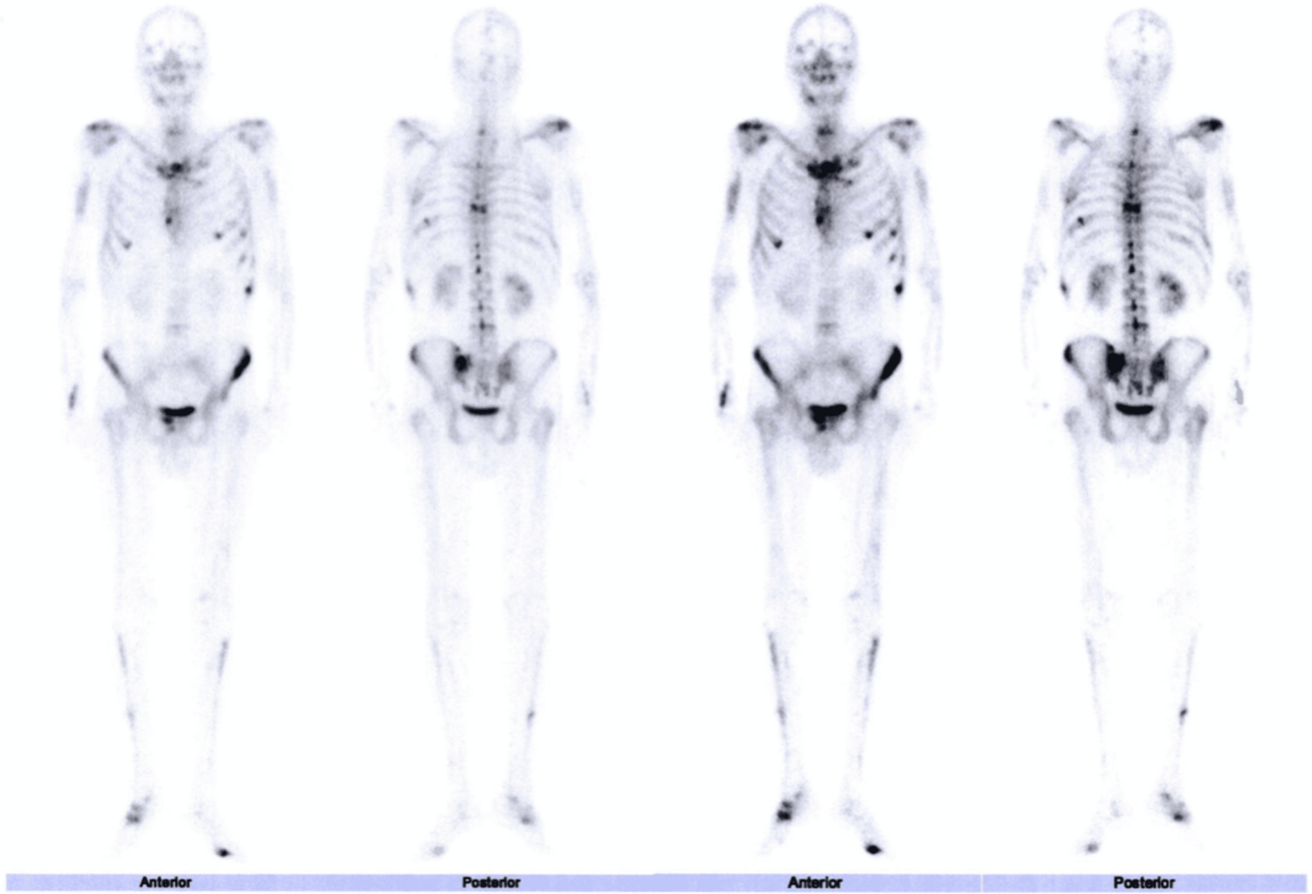
Bone scintigraphy The bone scintigraphy shows heterogeneous uptake in the skull, facial bones, jaw, entire vertebral axis, ribs, pelvis, as well as lesser expression in the long bones of the upper and lower limbs

A bone scintigraphy (Figure 3) showed heterogeneous uptake in the skull, facial bones (with reinforcement in the orbital region), mandible, entire vertebral column, rib cage, pelvis (more pronounced on the left), and minimal expression in the long bones of the upper and lower limbs (including the right tarsus), with alterations of unclear etiology.

Since multiple myeloma was the main differential diagnosis for these osteolytic lesions with concomitant hypercalcemia, a bone biopsy was performed at a lesion site in the sternal region to rule it out. The histopathological examination revealed fragments of a lymphoid proliferation of large cells, with an immunohistochemical profile positive for CD20, CD10, BCL6, BCL2, and MYC and negative for MUM1, with a proliferative index (Ki67) of 40%-50%. The diagnosis was diffuse large B-cell lymphoma.

Once solid neoplasms with bone metastasis and visceral involvement of lymphoma were excluded, the diagnosis was confirmed as PBL. The patient showed favorable clinical and analytical evolution, with adjustment of analgesic therapy achieving pain control. At the time of discharge, renal function was normalized, and hypercalcemia had resolved. Following the diagnosis, the patient was referred to hematology outpatient care and received chemotherapy, with a good response during the first few months. However, due to an infectious complication requiring intensive care unit admission, the patient passed away one year after the diagnosis.

## Discussion

PLB was first described by Oberling in 1928 [7], and in 1939, Jackson and Parker reported the first case series of 17 patients with primary reticulum cell sarcoma of bone or, as it is now known, PLB. The majority are diagnosed as diffuse large B-cell lymphoma. Other subtypes found in a minority of cases include follicular lymphoma, small lymphocytic lymphoma, marginal zone lymphoma, anaplastic large cell, and Burkitt's lymphoma. Any part of the skeleton can be involved [8]; however, the long bones are more affected, with the femur being the most common site. The pelvis is the second most affected site, while other localizations include the spine, ribs, mandible, scapula, and proximal phalanx of the thumb [9]. 

Although PLB has been an established clinical entity for more than six decades, its definition, diagnosis, and treatment remain controversial. This clinical case was a diagnostic challenge, and one of the important reasons for this write-up is to advocate for keeping the list of possible differential diagnoses, as it had a typical clinical presentation of multiple myeloma with hypercalcemia, renal failure, and osteolytic lesions. However, in the absence of elevated free light chains or monoclonal gammopathy and the exclusion of plasma cells in the bone marrow, it became necessary to proceed with an ultrasound-guided bone biopsy on a sternal lesion, leading to the final diagnosis.

## Conclusions

The differential diagnosis is essential for the correct management and prompt treatment of these patients, as this condition has a different treatment approach and a better prognosis than other bone tumors and even multiple myeloma itself. Thus, this case of multifocal PBL is presented, highlighting the challenge in diagnosing this entity, the need for a multidisciplinary approach in the management of the patient, and a high index of suspicion for this condition when diagnosing multiple osteolytic lesions.
